# PCSK6 mediates Th1 differentiation and promotes chronic colitis progression and mucosal barrier injury via STAT1

**DOI:** 10.18632/aging.204739

**Published:** 2023-05-20

**Authors:** Xiaoping Mei, Hongkun Zhou, Zhengwei Song, Xiaodan Yang, Xiaorong Liu, Jianguo Fei, Yiyu Shen, Xiaoguang Wang

**Affiliations:** 1 Department of Surgery, Affiliated Hospital of Jiaxing University, Jiaxing 314000, Zhejiang, People’s Republic of China; 2 Department of Surgery, The Second Affiliated Hospital of Jiaxing University, Jiaxing 314000, Zhejiang, People’s Republic of China

**Keywords:** PCSK6, chronic colitis, helper T cells, M1 macrophages, inflammation

## Abstract

This study was aimed at investigating the expression and role of proprotein convertase subtilisin/kexin type (PCSK6) in inflammatory bowel disease (IBD). DSS induced mouse colitis and mucosal barrier injury, down-regulation of TJ proteins, improvement of permeability, and increases of the proportions of Th1 and M1 macrophages. After PCSK6 knockdown, the colitis in KO mice was improved relative to WT mice, the TJ protein levels increased, and the proportions of Th1 and M1 macrophages decreased. STAT1 inhibitor treatment also inhibited chronic colitis in mice. As revealed by *in-vitro* experiments, PCSK6 overexpression promoted the transformation of Th0 into Th1, while PCSK6 silencing suppressed the transfection. COPI assay results revealed the presence of targeted binding relation between PCSK6 and STAT1.

PCSK6 binds to STAT1 to promote STAT1 phosphorylation and regulate Th1 cell differentiation, thus promoting the M1 polarization of macrophages and aggravating colitis progression. PCSK6 is promising as the new target for the treatment of colitis.

## INTRODUCTION

PCSK6 is a kind of calcium-dependent subtilase-like endonuclease with secretory property, which can be persistently secreted outside the cells [1]. As a kind of intercellular substance binding protein, PCSK6 is located on the cell membrane via the cysteine-rich region and binds to the membrane-related substrates. In addition, it exerts its effect through the transformation of inactivated precursor substances like membrane binding proteins and transcription factors into the activated forms via the splitting action of conjugated amino acids [2], in other words, PCSK6 has activation regulatory effects. It was recently discovered in rheumatoid arthritis (RA) and atherosclerosis (AS) that, PCSK6 plays an important role [3], and high expression of PCSK6 can stimulate the occurrence and progression of inflammatory response [4, 5]. However, the level and role of PCSK6 in other diseases have not been reported, in other words, the research on PCSK6 is lacking a precedent. As discovered by protein interaction prediction, PCSK6 may interact with STATs proteins, while the STATs family is related to the transformation of multiple immune cells [6, 7]. It was discovered in inflammatory bowel disease (IBD) research that, PCSK6 expression increases in IBD, meanwhile, PCSK6 is closely related to the increased proportion of Th1 cells.

## MATERIALS AND METHODS

### Flow cytometry (FCM)

Detection of Th1 and Th2 cell proportions [8]: The separated PBMCs, which included FITC-CD4, PE-F4/80 and APC-IL-4 monoclonal antibodies (BD, MA, USA), were subjected to resuspension within 50 μl solution. Thereafter, the sample was added and detected, with results being displayed in %.

F4/80+CD11b+M1 cell proportion measurement: Briefly, cells were subjected to 20-min incubation using antibodies (10 μl), which includedFITC-CD11b and PE-F4/80 monoclonal antibodies (BD, MA, USA). Results were displayed in %.

### ELISA

For detecting cytokines in tissues, PBS was added to wash gut tissues twice, followed by liquid nitrogen grinding till no granule could be observed. Later, samples were lysed with NP-40 lysate (1 ml, Beyotime Biotechnology Co., Ltd, Shanghai, China) for a 30-min period on ice, followed by collection of supernatant protein liquid. Cell experiments were conducted to measure cytokine contents within the medium. To be specific, the isolated medium was stored under -80^°^ C, samples obtained were later subjected to 20-min centrifugation for detection in line with ELISA kit (Jiancheng Institute of Biology, Nanjing, China) protocols, and calculated by standard curve approach, with results being displayed in ng/ml.

### Mouse grouping and chronic colitis model establishment

This work built the chronic colitis mouse model through using disuccinimide octanediate (DSS) [9]. Female wild-type (WT) C57BL/6 mice (weight, 21-25 g), and female PSCK6 knockdown PCSK6^-/-^ (KO) mice (Cyagen Company, Suzhou, China; weight, 21-25 g) were adapted to environment for one week and later utilized in this experiment. These animals were later classified as WT, KO, WT-DSS or KO-DSS group (n=10 each). For those of DSS group, they were fed with 2.5% DSS for constructing the chronic colitis mouse model. Specifically, 2.5% DSS was administered on days 1-5, 8-12, 15-19 and 22-26, while purified water was provided on rest days in each mouse.

To explore the underlying mechanism, mice in WT group were further classified as Control, DSS, and DSS+Cirsilineol groups. Cirsilineol, the STAT1 inhibitor, was intraperitoneally injected at 0.5 mg/kg/Day to block the STAT1 signal. Remaining treatments were the same as aforementioned descriptions.

### FITC-D-based detection of mucosal barrier permeability

Fluorescein isothiocyanate-dextran (FITC-D) was administered into each mouse via gavage, then its level was detected, and alterations of intestinal mucosal permeability was judged. Specifically, following mouse intervention, mice were fasted for water and food for a 4-h period before they were killed. Later, FITC-D (MW=400, 60 mg FITC-D/100 g) was given into each mouse through gavage, then FITC-D content in serum was detected, and a fluorescence spectrophotometer was utilized to analyze the sample fluorescence density.

### Western-blot (WB) analysis

Protein content was measured, then samples were subjected to electrophoresis for protein separation, followed by transfer on PVDF membrane. Thereafter, 5% defatted milk was added to block membranes for a 2-h period, prior to overnight incubation using TBST-diluted primary monoclonal antibodies (dilution, 1:300-1:500; Abcam, MA, USA) under 4^°^ C. Afterwards, HRP-labeled IgG secondary antibody (1:2000; Abcam, MA, USA) was further added for membrane incubation. Then, chemiluminescence was conducted to detect protein bands, while Image Pro-Plus 6.0 software was employed to analyze absorbance (OD), and GAPDH was an internal control. Results represented target protein-to-control protein OD ratio.

### Cell experiments

Th0 cells were transfected with PCSK6 overexpression plasmid (pLV2-CMV-PCSK6, Genepharma Company, Shanghai, China) for constructing PCSK6-OV Th0 cells. Thereafter, siRNA was used to silence PCSK6 expression, and the transiently transfected siRNA concentration was 160 nmol/L. Th0 cells were seeded in 12-well plates at the density of 2×10^5^ cells/well, and transient transfection was conducted according to the HiPerFect transfection reagent (Qiagen Company, Germany) instructions to obtain the PCSK6-Si Th0 cells.

### Statistical analysis

All data were represented by (±s). Data in two groups were compared by two independent sample t-test, while those across multiple groups by one-way ANOVA. P<0.05 (two-sided) was indicative of significant difference.

### Data availability statement

The data supporting the findings of this study are available from the corresponding author upon reasonable request.

## RESULTS

### PCSK6 aggravated mouse intestinal inflammation and increased the proportions of Th1 and M1 macrophages

**Figure 1:**
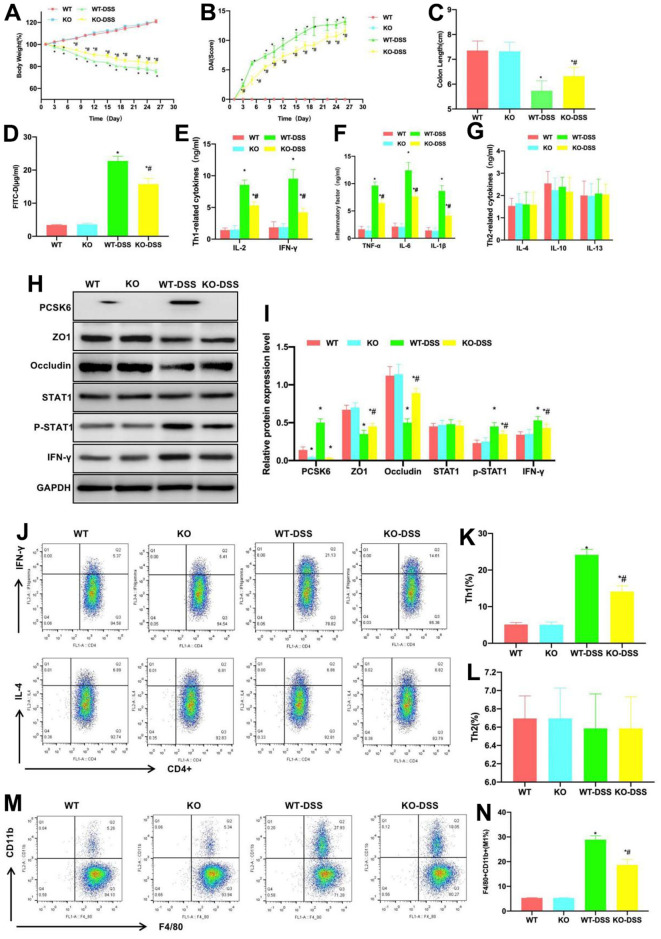
**Effect of PCSK6 on mice with colitis.** (**A**) Dynamic measurement of mouse body weight (n=10). Body weights of WT and KO mice gradually increased, while those of KO-DSS and WT-DSS groups gradually decreased, but KO-DSS group had notably less decreases relative to WT-DSS group, with difference being of significance. ^*^P<0.05 vs. WT group, ^#^P<0.05 vs. WT-DSS group. (**B**) DAI dynamic scores (n=10). The DAI scores of KO-DSS and WT-DSS groups gradually increased, but those in WT and KO remained largely unchanged, and the score of KO-DSS group was lower than WT-DSS group at corresponding time point, with significant difference. ^*^P<0.05 vs. WT group, ^#^P<0.05 vs. WT-DSS group. (**C**) Comparison of mouse intestinal length (n=10). Intestinal tissue lengths in KO-DSS and WT-DSS groups decreased relative to WT and KO groups, but KO-DSS group had increased length relative to WT-DSS group. ^*^P<0.05 vs. WT group, ^#^P<0.05 vs. WT-DSS group. (**D**) FITC-D (n=10). Serum FITC-D contents in WT and KO mice were lower, while those of WT-DSS and KO-DSS mice significantly increased, and those of KO-DSS mice decreased relative to WT-DSS mice. ^*^P<0.05 in relative to WT group, ^#^P<0.05 in relative to WT-DSS group. (**E**–**G**) ELISA (n=10). IL-6, IFN-γ, IL-4, TNF-α and IL-1β contents of WT and KO groups were lower, while these contents in KO-DSS and WT-DSS mice significantly increased compared with WT and KO mice, but their contents within KO-DSS mice decreased relative to WT-DSS mice. Differences in the Th2 cells-related factors among the four groups were not significant. ^*^P<0.05 vs. WT group, ^#^P<0.05 vs. WT-DSS group. (**H**, **I**) Relative protein level (n=5). PCSK6 was not significantly expressed in KO or KO-DSS group. ZO1 and Occludin expression of KO and WT groups increased, and their levels of KO-DSS and WT-DSS groups were markedly down-regulated. p-STAT1 expression in KO-DSS mice notably decreased relative to WT-DSS mice. ^*^P<0.05 vs. WT group, ^#^P<0.05 vs. WT-DSS group. (**J**–**N**) Th1, Th2 cells and M1 macrophages did not exhibit significant differences in WT versus KO groups, and low proportions could be detected, while Th1 cells and M1 macrophages proportions in WT-DSS and KO-DSS groups significantly increased relative to WT and KO groups. However, Th2 cell proportion remained largely unchanged, while Th1 cells and M1 macrophages proportions of KO-DSS group decreased compared with WT-DSS group. ^*^P<0.05 vs. WT group, ^#^P<0.05 vs. WT-DSS group.

The WT and PCSK6^-/-^ knockdown KO mice were utilized for experiments. DSS induced the occurrence of mouse chronic colitis. According to dynamic detection of body weight, the body weights of WT and KO mice gradually increased, and there existed no significant difference between groups. By contrast, the body weights of KO-DSS and WT-DSS mice gradually decreased, but the decrease level in KO-DSS group significantly reduced in relative to WT-DSS group, and the difference was of statistical significance (Figure 1A). DAI scores showed that, the scores of KO-DSS and WT-DSS groups gradually increased, while those in WT and KO groups remained largely unchanged, but the score of KO-DSS group at the same time point was lower than that of WT-DSS group, and the difference was of significance (Figure 1B). The mouse intestinal tissue length was also measured, which suggested that the intestinal tissue lengths in KO-DSS and WT-DSS groups were lower than those in WT and KO groups, but that in KO-DSS group was longer than that in WT-DSS group (Figure 1C). As discovered in detecting mucosal barrier permeability, the serum FITD-D levels in WT and KO mice were lower, with a lower permeation rate, whereas the levels in WT-DSS and KO-DSS groups significantly increased, and that in KO-DSS group was lower than that in WT-DSS group (Figure 1D). In cytokine detection, the levels of Th1 cell markers and inflammatory factors IL-6, IFN-γ, IL-4, TNF-α and IL-1β in WT and KO groups were lower, while those in KO-DSS and WT-DSS groups significantly increased, higher than those of WT and KO groups, but the levels in KO-DSS group were lower than those in WT-DSS group. The differences in Th2 cells-related factors were not notable among the four groups (Figure 1E–1G). In protein detection, we discovered that PCSK6 was significantly knocked down in KO and KO-DSS groups. TJ proteins (ZO1 and Occludin) were highly expressed in KO and WT groups, their levels in KO-DSS and WT-DSS groups significantly decreased, and the levels in KO-DSS group were higher than those in WT-DSS group. The STAT1 phosphorylation level elevated under the induction of DSS, but the p-STAT1 level in KO-DSS group was apparently lower than that in WT-DSS group (Figure 1H, 1I).

As discovered in the detection of Th1/Th2 cells and M1 macrophages, the proportions of Th1 and Th2 cells and M1 macrophages in WT and KO groups were low, and the differences were not significantly different. Besides, the proportions of Th1 cells and M1 macrophages notably increased in WT-DSS and KO-DSS groups, higher than those in WT and KO groups, whereas the proportion of Th2 cells was not apparently changed. Therefore, Th1 cells and M1 macrophages were dominant in intestinal inflammation, but their proportions in KO-DSS group were lower than those in WT-DSS group (Figure 1J–1N).

### Suppressing STAT1 antagonized the effect of PCSK6, alleviated mouse intestinal inflammation, and decreased the proportions of Th1 cells and M1 macrophages

**Figure 2:**
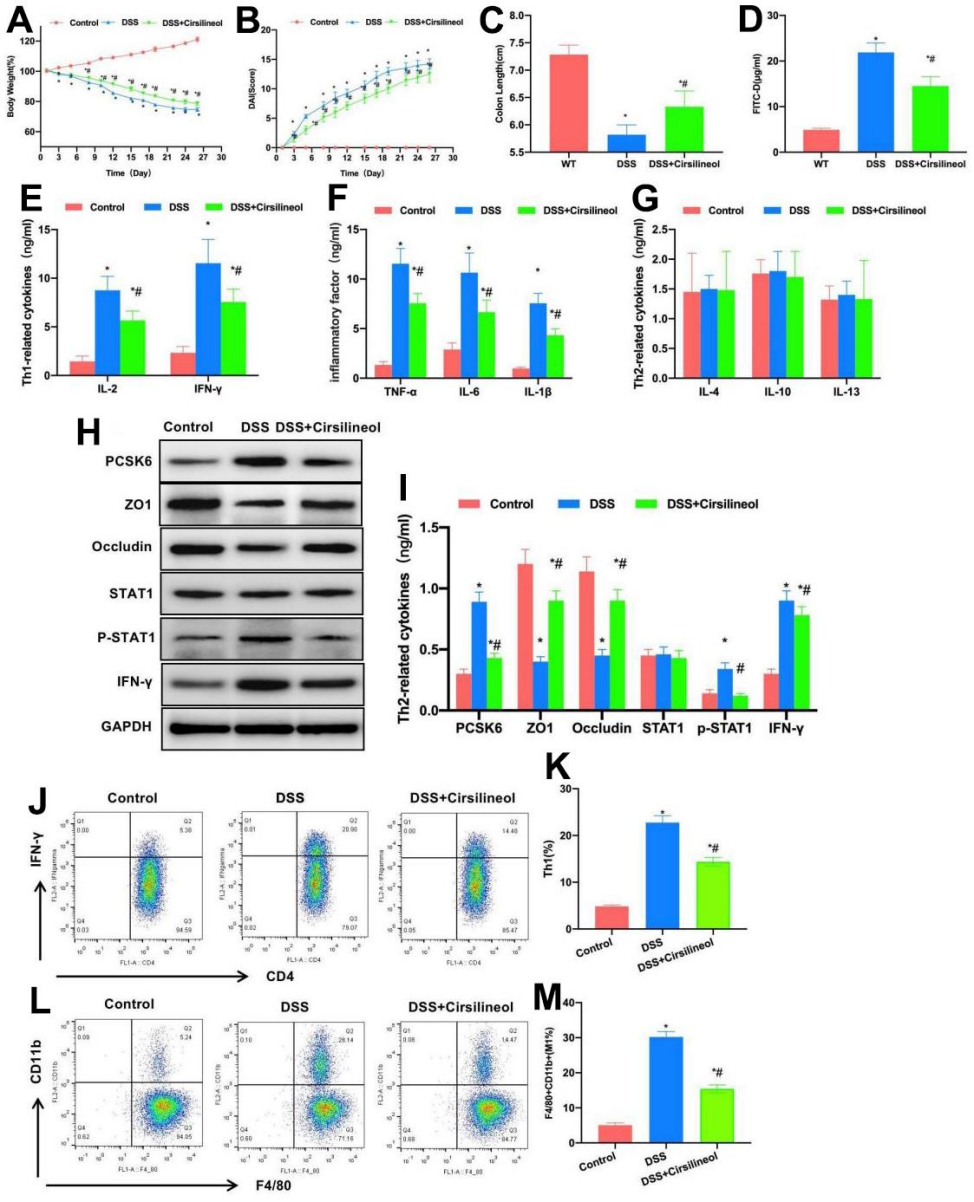
**Role of suppressing STAT1 in mouse colitis.** (**A**) Dynamic measurement of mouse body weight (n=10). Weight loss in mice of Cirsilineol group was mitigated relative to DSS group. ^*^P<0.05 vs. Control group, ^#^P<0.05 vs. DSS group. (**B**) DAI dynamic score (n=10). The DAI score of Cirsilineol group was lower than that of DSS group. ^*^P<0.05 in relative to Control group, ^#^P<0.05 in relative to DSS group. (**C**) Mouse intestinal length (n=10). Cirsilineol group had increased intestinal length compared with DSS group. ^*^P<0.05 vs. Control group, ^#^P<0.05 vs. DSS group. (**D**) FITC-D (n=10). Cirsilineol reduced serum FITC-D content, with significant difference relative to DSS group. ^*^P<0.05 in relative to Control group, ^#^P<0.05 in relative to DSS group. (**E**–**G**) ELISA (n=10). Cirsilineol decreased IL-2, IFN-γ, TNF-α, IL-6 and IL-1β contents in tissues, while IL-4, IL-10 and IL-13 (markers of Th2 cells) expression remained largely unchanged. ^*^P<0.05 vs. Control group, ^#^P<0.05 vs. DSS group. (**H**, **I**) Relative protein expression levels (n=5). Cirsilineol increased tissue TJ protein levels, but decreased the PCSK6 as well as p-STAT1 levels. ^*^P<0.05 vs. Control group, ^#^P<0.05 vs. DSS group. (**J**–**M**) Relative to DSS group, Th1 cells and M1 macrophages proportions of Cirsilineol group remarkably declined, with significant differences. ^*^P<0.05 vs. Control group, ^#^P<0.05 vs. DSS group.

STAT1 signal is the major signal of Th1 differentiation. In this experiment, STAT1 inhibitor was used to block the STAT1 signal in WT mice, which suppressed Th1 formation and intestinal inflammation. As discovered in the dynamic detection of mouse body weight, the body weight loss in Cirsilineol group was alleviated in comparison with DSS group, and Cirsilineol suppressed mouse body weight loss (Figure 2A). Moreover, as discovered in DAI score detection, the mouse DAI score in Cirsilineol group was lower than that in DSS group, and the difference was significant (Figure 2B). Based on mouse intestinal length detection, the intestinal length of mice after Cirsilineol administration was longer than that of DSS group (Figure 2C). According to FITC-D detection, Cirsilineol reduced the serum FITC-D level, and the difference was statistically significant in relative to DSS group (Figure 2D). In Th1 cell markers and inflammatory factors detection, compared with DSS group, Cirsilineol reduced the levels of IL-2, IFN-γ, TNF-α, IL-6 and IL-1β in tissues, while those of Th2 cell markers IL-4, IL-10 and IL-13 were not notably changed (Figure 2E–2G). In addition, protein detection also indicated that, Cirsilineol increased the TJ protein levels in tissues, reduced the PCSK6 and p-STAT1 expression, and the differences were significant in comparison with DSS group (Figure 2H, 2I).

FCM results also suggested that, compared with DSS group, the proportions of Th1 cells and M1 macrophages in Cirsilineol group significantly decreased (Figure 2J–2M).

### Effect of PCSK6 *in vitro* on Th1 formation

**Figure 3:**
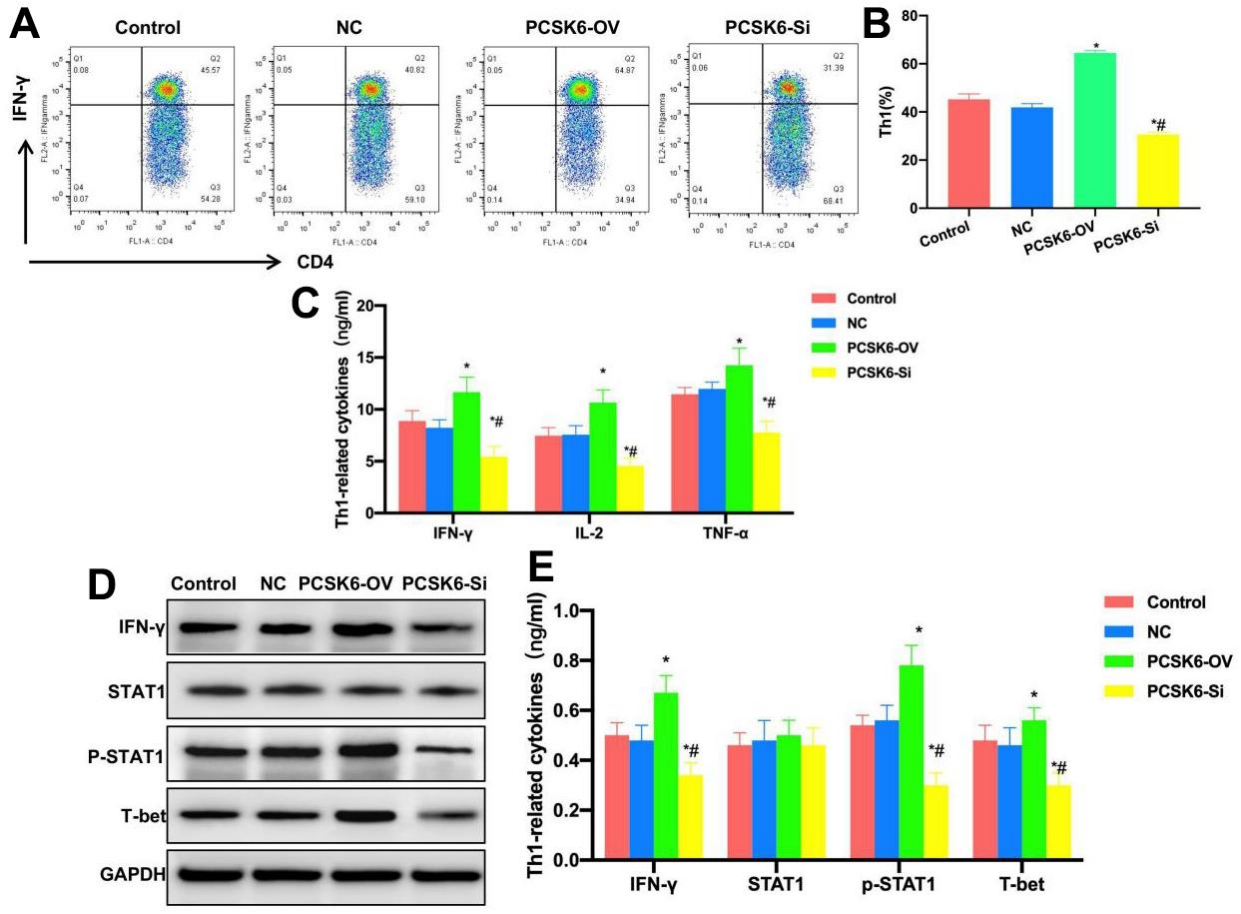
**Role of PCSK6 in Th1 cell differentiation.** (**A**, **B**) FCM (n=3). The proportion of Th1 cells did not show significant difference in Control vs. NC groups, that in PCSK6-OV group significantly increased relative to Control group, and that of PCSK6-Si group decreased relative to Control group. ^*^P<0.05 in relative to Control group, ^#^P<0.05 in relative to NC group. (**C**) ELISA (n=3). IL-2, TNF-α and IFN-γ contents of PCSK6-OV group significantly increased relative to those in Control group, but their contents of PCSK6-Si group decreased relative to Control group. ^*^P<0.05 vs. Control group, ^#^P<0.05 vs. NC group. (**D**, **E**) Relative protein expression levels (n=3). p-STAT1 and T-bet contents of PCSK6-OV group increased, while p-STAT1 level decreased in PCSK6-Si group, with significant difference relative to Control group. ^*^P<0.05 vs. Control group, ^#^P<0.05 vs. NC group.

This work investigated the effect of PCSK6 *in vitro* on the differentiation of Th0 cells into Th1 cells. To be specific, PCSK6 was over-expressed (PCSK6-OV) and silenced (PCSK6-Si) in Th0 cells. After induction, the proportion of Th1 cells between Control group and NC group was not significantly different, that in PCSK6-OV group significantly increased, higher than that in Control group, whereas that in PCSK6-Si group was lower than that in Control group, and the difference was significant (Figure 3A, 3B). According to marker cytokine detection, the levels of IL-2, TNF-α and IFN-γ in PCSK6-OV group were significantly up-regulated relative to Control groups, while those in PCSK6-Si group were lower than those in Control group (Figure 3C). According to protein detection results, STAT1 was activated in PCSK6-OV group, the p-STAT1 level was up-regulated, the transcription factor T-bet level increased, but the p-STAT1 level decreased in PCSK6-Si group, and the difference was of significance relative to Control group (Figure 3D, 3E).

### Suppressing STAT1 inhibited Th1 cell differentiation

**Figure 4:**
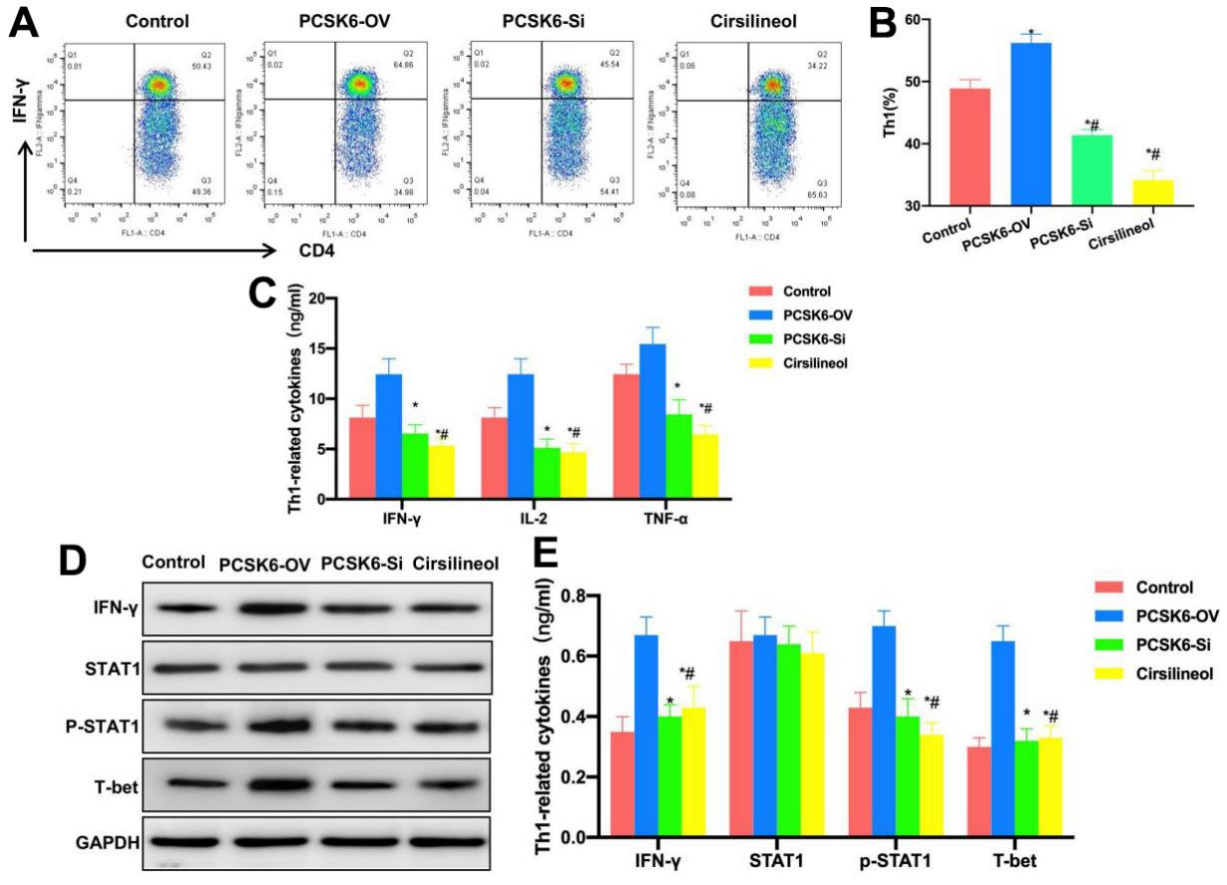
**Suppressing STAT1 inhibited Th1 cell differentiation.** (**A**, **B**) FCM (n=3). Cirsilineol suppressed Th1 cell differentiation. The Th1 cells proportion remarkably decreased compared with those of PCSK6-OV, PCSK6-Si and Control groups. ^*^P<0.05 vs. Control group, ^#^P<0.05 vs. PCSK6-OV group. (**C**) ELISA (n=3). IL-2, IFN-γ, TNF-α, IL-6 and IL-1β contents of Cirsilineol group decreased relative to Control and PCSK6-OV groups, with no significant difference versus PCSK6-Si group. ^*^P<0.05 vs. Control group, ^#^P<0.05 vs. PCSK6-OV group. (**D**, **E**) Relative protein expression (n=3). IFN-γ, p-STAT1 and T-bet levels in Cirsilineol group remarkably decreased relative to PCSK6-OV group, with no significant difference versus PCSK6-Si group. ^*^P<0.05 vs. Control group, ^#^P<0.05 vs. PCSK6-OV group.

Cells were treated with STAT1 inhibitor, compared with siRNA treatment, Cirsilineol also inhibited Th1 cell differentiation. The Th1 cell proportion was significantly lower than those in PCSK6-OV, PCSK6-Si and Control groups (Figure 4A, 4B). Cytokine detection results also revealed that, the levels of IL-2, IFN-γ, TNF-α, IL-6 and IL-1β in Cirsilineol group were lower than those in Control and PCSK6-OV groups, but the difference was not significant in relative to PCSK6-Si group (Figure 4C). Relative protein expression detection also indicated that, the p-STAT1 level decreased in Cirsilineol group, which decreased T-bet expression accordingly (Figure 4D, 4E).

### Verification of the interaction between PCSK6 and STAT1

**Figure 5:**
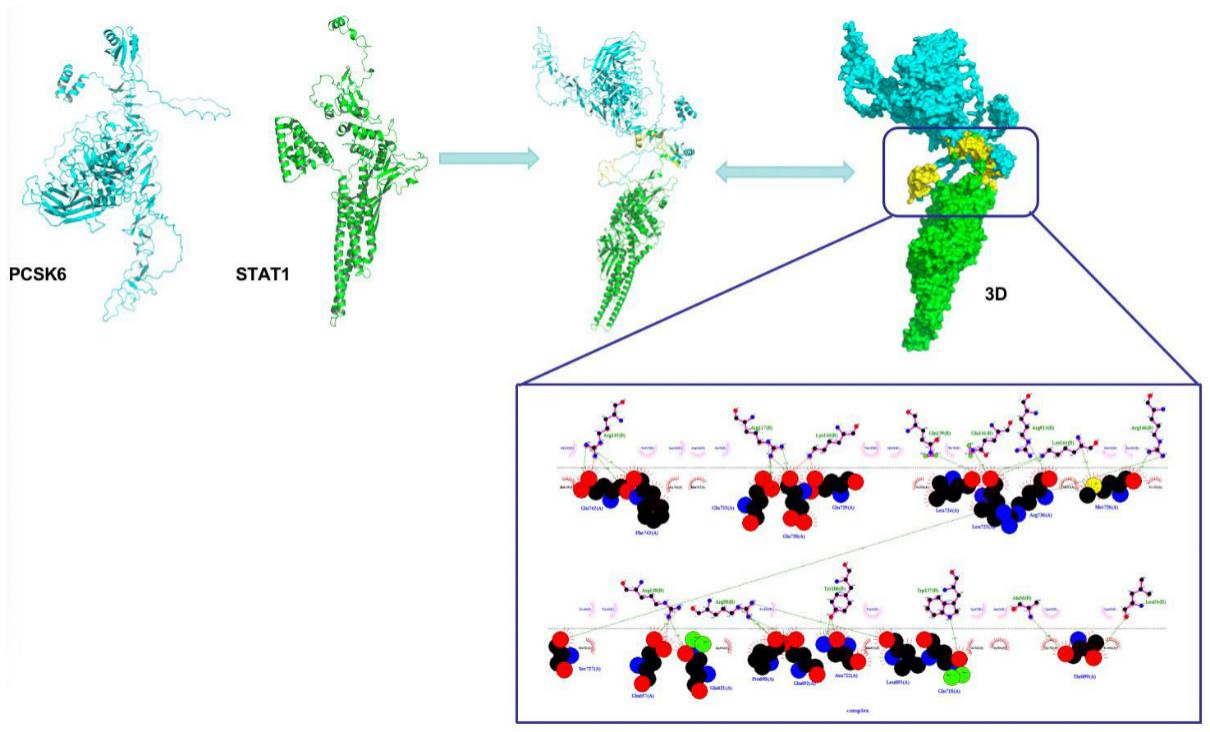
Interaction between PCSK6 with STAT1.

As discovered from protein-protein interaction analysis, there was binding relation between PCSK6 and STAT1. STAT1 is a protein distributed on cell membrane, which is activated after phosphorylation modification at tyrosine or serine locus. Our simulation results revealed that PCSK6 bound to the tyrosine locus, which was related to the STAT1 phosphorylation activation. (Figure 5).

## DISCUSSION

T helper (Th) lymphocytes are the important component of the human immune system, making an essential role in the immunity-mediated inflammatory diseases [10, 11]. Previous studies have discovered that peripheral inflammation is related to the peripheral T lymphocytes-secreted cytokines [12, 13]. Th1 and Th2 are the two important subsets of Th cells, Th1 cells mainly secrete the pro-inflammatory cytokines to participate in macrophage activation for mediating cellular immunity [14, 15], such as interleukin-6 (IL-6) [16], Interferon-γ (IFN-γ) [17]. By contrast, Th2 cells mainly secrete inhibitory cytokines like IL-4 and IL-10 [18, 19] to mediate humoral immunity. The functional regulation of Th1 and Th2 cells interacts with that of macrophages. IFN-γ generated by Th1 can promote macrophage transformation and reduce the cytophagic effect, thereby producing massive cytotoxic M1 macrophages [20, 21].

PCSK6 protein is a common homologous protein of PCSK9, both of which belong to the PCSKs protein family. PCSK6 is extensively expressed in numerous tissues, including neuroendocrine, liver, intestinal and the brain tissues [22]. At present, research on the role of PCSK6 is lacking, and this study attempted to reveal the role of PCSK6 in inflammatory disease with the IBD model. As discovered from human sample detection, PCSK6 expression significantly increased in the tissues of IBD patients, at the same time, the expression of PCSK6 was positively related to that of Th1 and Th1 cytokines IL-2, IFN-γ and TNF-α, but was not significantly associated with Th2. More importantly, Th1 cells are dominant in IBD, which are considered as the important pro-inflammatory T-cells in IBD. Administration of low concentration of DSS induced chronic colitis in mice, from our research results, mice exhibited obvious body weight loss, increased DAI score, inflammatory response, edema, and severe villous-like structural injury. The PCSK6-KO mice were compared with WT mice, as a result, after PCSK6 knockdown, the mouse intestinal inflammation was apparently alleviated, the DAI score decreased, and intestinal inflammation and edema were alleviated. Meanwhile, the TJ protein expression was up-regulated. TJ proteins are the proteins maintaining tight junction and arrangement of intestinal cells [23], their high expression levels indicate the intact mucosal barrier between intestinal cells and reduced permeability, while their down-regulation indicates the damage of the barrier. From our results, after intragastric administration of FITC-D in colitis mice, the serum FITC-D level significantly increased and the permeability elevated, while PCSK6 knockdown reduced the mucosal barrier permeability and serum FITC-D levels. Importantly, PCSK6 knockdown reduced the proportions of Th1 cells and M1 macrophages in tissues, thus, PCSK6 was speculated as the protein promoting Th1 differentiation. The mouse intestinal Th0 cells were separated, then PCSK6 expression was over-expressed/silenced in Th0 cells to induce their differentiation *in vitro*. As a result, PCSK6 overexpression promoted Th1 formation, and increased Th1 cell proportion, while PCSK6 silencing decreased Th1 cell proportion. Additionally, PCSK6 promoted STAT1 phosphorylation. As observed from previous studies, STAT1 is the key signal promoting Th1 formation [24, 25], which is subject to phosphorylation modification. P-STAT1 is the activated STAT1, which is adopted to activate the downstream signal, while PCSK6 mainly promotes the STAT1 phosphorylation level. Protein interaction simulation suggested that, PCSK6 bound to the STAT1 tyrosine locus, our COIP results revealed the binding relation between PCSK6 and STAT1. Therefore, we considered that PCSK6 mainly promoted STAT1 phosphorylation to exert its effect. To verify the effect of STAT1, the STAT1 inhibitor was used in animal experiments. Similarly, Cirsilineol also reduced the proportions of Th1 cells and M1 macrophages, and improved the mouse tissue inflammation, thereby down-regulating PCSK6 expression. According to TEM results, Cirsilineol also alleviated the mucosal barrier injury and villous-like structural injury. *In-vitro* experiments also suggested that, Cirsilineol also reduced Th1 formation, similar to the effect of siRNA-PCSK6.

## CONCLUSIONS

PCSK6 binds to STAT1 to promote the phosphorylation level of STAT1. In IBD, PCSK6 promotes Th1 cell formation via p-STAT1, thereby indirectly promoting the M1 polarization of macrophages. PCSK6 has an important effect on IBD, and PCSK6-STAT1-Th1 is promising as a new signal of IBD treatment, while the extensive role of PCSK6 in inflammatory disease deserves further investigation.
